# The new German evidence-based guideline on diffuse large B-cell lymphoma—key aspects for radiation oncologists

**DOI:** 10.1007/s00066-022-02035-9

**Published:** 2023-01-04

**Authors:** Michael Oertel, Christian Berdel, Gerhard Held, Klaus Herfarth, Heinz Schmidberger, Moritz Ernst, Georg Lenz, Peter Borchmann, Hans Theodor Eich

**Affiliations:** 1 Department of Radiation Oncology, University Hospital Muenster, Albert-Schweitzer-Campus 1, building A1, 48149 Muenster, Germany; 2 Department of Radiation Oncology, Saarland University Hospital, Homburg, Germany; 3 Department of Hematology/Oncology, Westpfalz-Klinikum, Kaiserslautern, Germany; 4 Department of Radiation Oncology, Heidelberg University Hospital, Heidelberg, Germany; 5 Department of Radiotherapy and Radiation Oncology, University Hospital Mainz, Mainz, Germany; 6 Evidence-based Oncology, Department I of Internal Medicine, Center for Integrated Oncology Aachen Bonn Cologne Duesseldorf, Faculty of Medicine and University Hospital Cologne, University of Cologne, Cologne, Germany; 7 Department of Medicine A, Hematology, Oncology, University Hospital Muenster, Muenster, Germany; 8 Department I of Internal Medicine, Center for Integrated Oncology Aachen Bonn Cologne Düsseldorf, University Hospital of Cologne, Cologne, Germany

**Keywords:** DLBCL, Radiotherapy, Aggressive lymphoma, Radiation oncology, Non-Hodgkin lymphoma

## Abstract

**Purpose:**

Diffuse large B‑cell lymphoma (DLBCL) is an aggressive lymphoma subtype treated successfully with immunochemotherapy. However, there are conflicting data on the role and impact of consolidative radiation therapy (RT). The publication of the national evidence-based guideline on DLBCL prompted us to review relevant passages on radiation oncology.

**Methods:**

The following article reviews the evidence and recommendations given in the current German evidence-based guideline on DLBCL regarding RT and summarizes pivotal aspects. Additional literature is presented to provide a comprehensive background for the published recommendations.

**Results:**

RT shall be administered to all patients with localized positron emission tomography(PET)-positive residues after completion of immunochemotherapy and should use a dose of 30–40 Gray in normofractionation. For RT planning, PET information before and after immunochemotherapy shall be used, with either a PET-CT in the RT treatment position or an image fusion to the planning CT. Conformal techniques shall be used for target volume coverage, with a risk–benefit evaluation for the individual patient. Additionally, RT may be used in the treatment context of various subtypes of DLBCL as well as in the recurrent or refractory treatment situation.

**Conclusion:**

RT remains an integral part of the treatment repertoire of DLBCL. With the use of PET-guided treatment, RT is indicated for patients with metabolically active tumors. In the context of the ongoing development of targeted therapies, new RT indications may evolve.

## Introduction

Diffuse large B‑cell lymphoma (DLBCL) is the most common aggressive non-Hodgkin lymphoma (NHL), constituting 31% of all NHL [1]. The current annual incidences are 29,100 cases in the United States and 26,000 in Western Europe, with a predicted increase in the next years [2]. Due to the otherwise dismal course of DLBCL, staging should be completed within a few weeks to initiate timely therapy thereafter. Immunochemotherapy with rituximab, cyclophosphamide, hydroxydaunorubicin, vincristine, and predniso(lo)ne (R-CHOP)-based regimens are the backbone of therapy to enable long-term cure, which is achieved in approximately 65% of patients [3]. However, the role of radiation therapy (RT) is yet to be defined and is subject to discussions.

During the past 2 years, experts in various disciplines such as hematology, pathology, radiology, and radiation oncology elaborated a common evidence-based guideline, which was published in June 2022 [4]. The project was funded and supported by the German Guideline Program in Oncology (registration no. 018-038OL).

The following article reviews important aspects for radiation oncologists.

## Radiotherapy in first-line treatment—PET-guided therapy takes center stage

Historically, the indication for RT was based on prechemotherapy morphological risk factors such as extranodal involvement or bulky disease. The UNFOLDER trial randomized patients aged 18–60 years with an age-adjusted international prognostic index of 0–1 between three-weekly R‑CHOP-21 and a two-weekly schedule (R-CHOP-14) [5]. A second randomization occurred after completion of immunochemotherapy between consolidative RT to bulky and extranodal sites with 39.6 Gray (Gy) versus observation (see Table 1 for an overview). Despite the improvement of the primary endpoint event-free survival (EFS) by means of RT (84% vs. 68%; *p* = 0.001), no significant changes in progression-free survival (PFS) or overall survival (OS) could be demonstrated (PFS: 89% vs. 81%; *p* = 0.221; OS: 93% vs. 93%, *p* = 0.506) in an interim analysis [5]. Final results of this trial are pending. The RICOVER-60 trial identified six cycles of R‑CHOP-14 (plus two cycles of rituximab) as an optimal treatment for patients aged 61–80 years and administered RT with 36 Gy to sites of initial bulky (≥ 7.5 cm) disease or extralymphatic involvement [6]. A comparison with a subsequent trial (RICOVER-noRTh) using the same immunochemotherapy without RT revealed an inferior EFS without RT in patients with bulky disease (40%; 95% confidence interval [CI]: 26–55% vs. 66%; 95% CI: 57–75%; *p* = 0.001), mainly due to unplanned RT series counting as an event [7]. Focusing on patients with bulky disease treated according to protocol, significant advantages in 3‑year EFS (54%; 95% CI: 38–71% vs. 80%; 95% CI: 71–89%; *p* = 0.001), PFS (62%; 95% CI: 46–78% vs. 88%; 95% CI: 80–95%; *p* < 0.001), and OS (65%; 95% CI: 49–81% vs. 90%; 95% CI: 84–97%; *p* = 0.001) were reported with the use of RT. Without an obligatory positron emission tomography (PET) scan after systemic therapy, the role of the metabolic status remains elusive.

**Table 1:** Pivotal trials investigating the use of radiotherapy in patients with DLBCL. Trial characteristics and outcomes are provided. Details on randomization focus on radiotherapy questions. Outcomes for the RICOVER-60 trial refer to the best arm of the 2×2 design (6×R-CHOP)

Study	Patients	*n*	RT indication	Randomization	Dose	EFS	PFS/TTP	OS	Key message
UNFOLDER ([5] interim analysis)	18–60 years aaIPI 0–1	285	Bulk ($\geq$7.5 cm) Extranodal	RT vs. no RT	39.6 Gy	3‑year: 68% vs. 84% (*p* = 0.001)	3‑year: 89% vs. 81% (n. s.)	3‑year: 93% vs. 93% (n. s.)	RT reduces PR rate triggering additional treatments
RICOVER-60 [6]	61–80 years	1222	Bulk ($\geq$7.5 cm) Extranodal	No RT randomization	36 Gy	3‑year: 66.5%	3‑year: 73.4%	3‑year: 78.1%	Establishment of 6×R-CHOP as standard of care
OPTIMAL>60 ([8] interim analysis)	61–80 years	187	PET-positive patients with initial bulk ($\geq$7.5 cm)	Comparison to RICOVER-60	39.6 Gy	–	2‑year: 79% vs. 75% (n. s.)	2‑year: 88% vs. 78% (n. s.)	RT may be limited to PET-positive patients
Freeman et al. [10]	$\geq$ 18 years Advanced stages	723	PET-positive patients (Deauville: 4–5) after 6–8 cycles of R‑CHOP	None Retrospective comparison	30–40 Gy	–	3‑year: 83% (PET-NEG) vs. 76% (PET-POS + RT; n. s.) vs. 34% (PET-POS-RT; *p* < 0.001)	3‑year: 87% (PET-NEG) vs. 80% (PET-POS + RT) vs. 44% (PET-POS-RT)	RT may be limited to PET-positive patients. Bulky disease ($\geq$ 10 cm), skeletal or craniofacial involvement were no risk factors independent from PET status

*aaIPI* age-adjusted international prognostic index, *EFS* event-free survival, *Gy* Gray, *n.* *s.* not significant, *OS* overall survival, *PET* positron emission tomography, *PET-NEG* PET-negative patients after immunochemotherapy, *PET-POS* *+* *RT* PET-positive patients after immunochemotherapy with consolidative radiotherapy, *PET-POS-RT* PET-positive patients after immunochemotherapy without consolidative radiotherapy, *PFS* progression-free survival, *R‑CHOP* rituximab, cyclophosphamide, doxorubicin, vincristine, predniso(lo)ne, *RT* radiotherapy, *TTP* time to progression

The present guideline follows the rationale of trials in the modern era by establishing a risk stratification via PET. Preliminary data from the OPTIMAL>60 study point towards the possibility of limiting RT to patients who are PET positive after systemic therapy [8, 9]. Patients treated in this study were 61–80 years old and randomization was undertaken after four cycles of immunochemotherapy with R‑CHOP or R‑CHLIP (liposomal vincristine instead of the conventional vincristine). Patients with a Deauville score of 3–5 were defined as PET positive and received two additional cycles of systemic therapy and involved-site radiotherapy of 39.6 Gy. This approach led to a 42% reduction of RT series without deterioration in PFS and OS as compared to RICOVER-60. Notably, only 22% of patients in the RT arm were not irradiated (mostly due to progression or medical reasons). Final results of this trial have not yet been published and are eagerly awaited.

In the absence of prospective evidence, the mainstay of PET-guided RT in DLBCL is a retrospective analysis from British Columbia [10]. In the work by Freeman and colleagues, 723 patients with a median age of 65 years were included, predominantly in Ann Arbor stage III or IV (74%) and with bulky disease in 39% of patients. RT was limited to patients with a Deauville score of 4 or 5 after at least six cycles of R‑CHOP. This approach could ameliorate the prognosis of patients with a positive end-of-treatment PET who underwent consolidative RT, reaching nearly the level of initial PET-negative patients (3-year time to progression: 76% vs. 83%; *p* = 0.3; 3‑year OS: 80% vs. 87% for the irradiated vs. initial PET-negative subgroups, respectively). Neither the initial presence of bulky disease, craniofacial involvement, nor skeletal manifestations heralded a worse time to progression. In summary, patients with localized PET-positive residual lymphoma after completion of immunochemotherapy shall receive consolidative RT.

## Radiotherapy planning

**Fig. 1:**
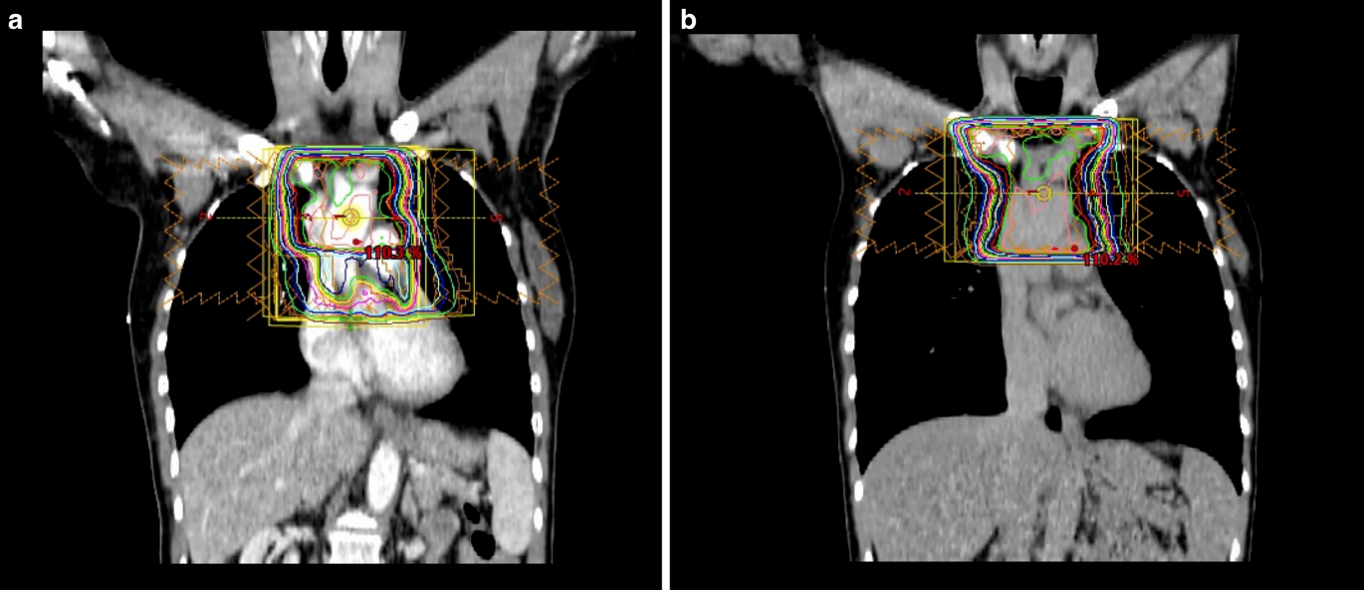
Deep inspiration breath-hold technique for mediastinal lymphoma. Frontal CT scans of the same patient with a mediastinal lymphoma residue prior to radiotherapy (RT) with isodose lines of a planned intensity-modulated RT (IMRT) plan. Scans were acquired in **a** free breathing and **b** deep inspiration breath-hold. Note the inflation of the lungs and the displacement of the heart from the target volume, enabling better sparing of these organs at risk

Implementation of PET guidance in the treatment algorithm offers additional information for RT planning. Either expansion of the target volume due to detection of additional lesions or adaption due to better differentiation between vital and avital residues may result. Consequently, existent PET examinations before and after immunochemotherapy shall be used for RT planning. This recommendation was formulated in analogy to the evidence-based guideline on Hodgkin lymphoma [11]. Data focusing on DLBCL patents are sparse; one analysis from the Memorial Sloan Kettering Cancer Center comparing CT-based vs. PET-CT-based contouring included 118 patients, but only 18 with DLBCL, the latter demonstrating a gross target volume expansion > 5% in 33% and a reduction > 5% in 28% with the use of PET [12]. As a result, the International Lymphoma Radiation Oncology Group (ILROG) also recommends the use of PET-CT, ideally performed in the treatment position or fused to the planning CT scan [13, 14]. In analogy to other mediastinal lymphomas (e.g., Hodgkin lymphoma), RT planning and execution in deep inspiration breath-hold technique should be evaluated (Fig. 1). Concerning the radiation plan, there is no general recommendation: a conformal technique shall be used with an individual benefit–risk evaluation of different approaches (3D conformal RT, intensity-modulated RT, volumetric modulated arc therapy). Among the techniques, proton therapy may be used to reduce the RT dose to organs at risk in the mediastinum, the impact of which on (long-term) toxicity is yet to be determined.

## Contouring

**Fig. 2:**
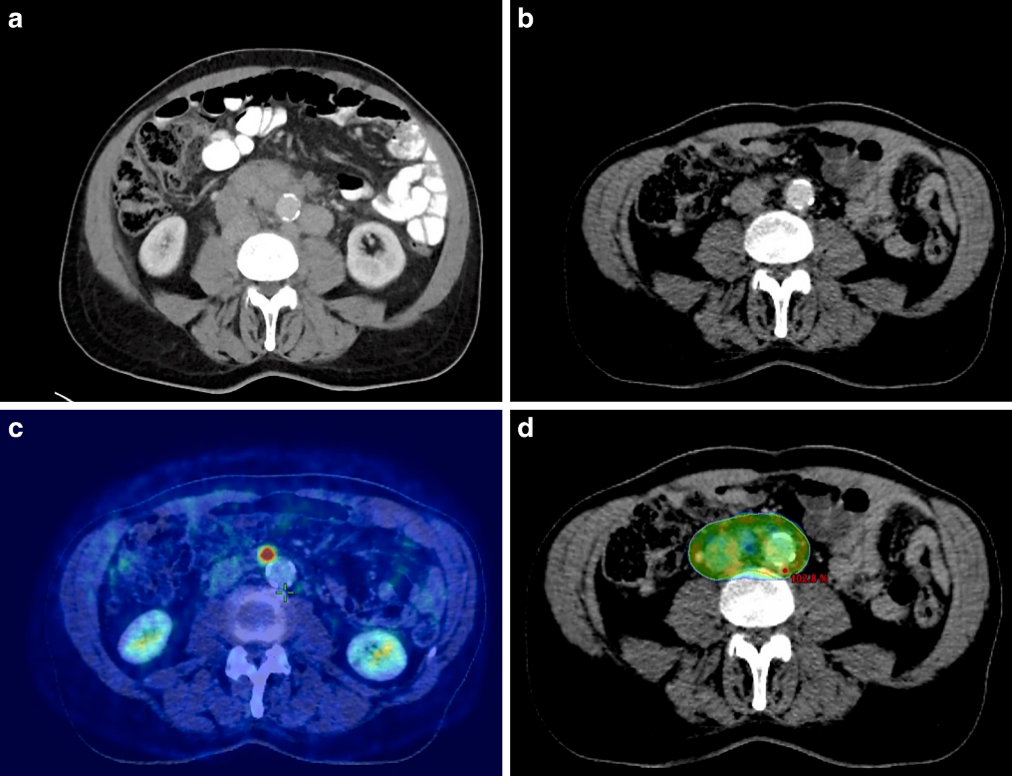
Consolidative radiotherapy of positron emission tomography (PET-)positive residues. **a** Initial (prechemotherapy) CT scan revealing an abdominal bulk of DLBCL. **b** Postchemotherapy CT scan of the abdomen showing an excellent response with only minimal residues. **c** F^18^-FDG-PET-CT indicates metabolically active tumor (Deauville 4) despite the good morphological response. **d** Colorwash representation of the 95% isodose covering the planning target volume (*thin orange line*). Radiation treatment was delivered via a volumetric modulated arc therapy photon plan

Modern RT planning shall use the concept of involved-site irradiation as introduced by the ILROG [13, 15]. The (sub)volumes used for RT planning are the gross tumor volume (GTV), clinical target volume (CTV), and planning target volume (PTV) with an optional internal target volume (ITV). The GTV covers macroscopic disease based on different examinations (PET, CT), whereas the CTV adapts the initial volume to the post-chemotherapy situation and adds an individual margin to account for subclinical spread (see Fig. 2). Importantly, the CTV has to include the post-chemotherapy (macroscopic) remaining lymphoma tissue. An ITV is essential for mobile target volumes in the thorax and abdomen and is established via 4D-CT or fluoroscopy. Otherwise, an isotropic margin of 1.5–2 cm may be used. Final expansions are introduced to consider positional uncertainties to receive the PTV.

Recent randomized trials on DLBCL such as MInT, RICOVER-60, UNFOLDER, and OPTIMAL > 60 utilize RT doses between 36 and 40 Gy in normofractionation [5, 6, 8, 9, 16], which may be seen as standard. However, a dose de-escalation study from the United Kingdom compared 30 Gy with 40–45 Gy as involved-field therapy for the treatment of aggressive lymphoma and failed to demonstrate differences in the rate of in-field recurrences (HR: 0.98; 95% CI: 0.68–1.4; *p* = 0.89) [17]. Overall, there are some limitations of this analysis, as the patient collective was mixed, covering treatments in the primary or recurrent situation and with curative as well as palliative intent. Radiological response assessment was not mandatory and no data on PET or morphological risk factors (e.g., bulk) were provided.

## Specific subtypes: extranodal involvement of bone, breast, stomach, testis, and primary mediastinal B-cell lymphoma

Apart from nodal lymphoma, RT may be used for the treatment of various subtypes and extranodal sites. In some cases (involvement of bones, primary mediastinal B‑cell lymphoma, primary extranodal DLBCL of the breast, gastric DLBCL without indolent component) the recommendation follows the algorithm of the PET-guided strategy described earlier. This is of particular importance for osseous involvement, which has been considered as a standard indication for RT due to the results of a pooled meta-analysis by the German High-Grade Non-Hodgkin’s Lymphoma Study Group [18]. The addition of RT after R‑CHOP immunochemotherapy led to a significant amelioration of 3‑year EFS (75% vs. 36%, *p* = 0.001). In the study by Freeman et al. (s. “Radiotherapy in first-line treatment”), 142 patients revealed skeletal involvement, 73% of whom became PET negative after R‑CHOP [10]. In patients with initial skeletal involvement, the rate of PET-negativity after systemic therapy did not differ from the overall study population (*p* = 0.8) and there was no significant difference in the time to progression compared to PET-negative patients without skeletal manifestations of lymphoma.

An important differentiation is described for gastric lymphoma, as patients with an indolent component (present in approximately one third of aggressive lymphomas of the stomach [19]) should be offered consolidative RT without consideration of the PET status. This rationale is supported by a Korean recurrence analysis, showing local or combined relapses as the principle location of treatment failure [20].

Patients with primary testicular DLBCL should receive a unilateral orchiectomy and shall be treated with subsequent R‑CHOP chemotherapy in analogy to nodal DLBCL. RT should be administered prophylactically to the contralateral testis after completion of systemic therapy, also including the ipsilateral testis, if no orchiectomy has been performed. In this context, RT results in a significant improvement in local control [21, 22], with one retrospective analysis postulating even an OS benefit [23].

## Recurrences/refractory disease

Regarding second-line therapy, a recommendation to irradiate patients with localized PET-positive residues after systemic therapy is given in the guideline, although there are no randomized studies addressing this aspect. Importantly, patients in the second-line situation who are unable/unfit to undergo systemic therapy shall be evaluated for palliative RT according to the respective ILROG guidelines [24]. The cited paper delineates different clinical scenarios for the refractory/recurrent situation, with a possible dose escalation beyond 40 Gy (up to 55 Gy). Additionally, RT shall be evaluated (beside chemo-, immuno-, or targeted therapy) in the setting of ≥ second recurrences or progression in a palliative setting or as a modality for remission induction before a curative (intended) therapy (“bridging” concept). For patients with primary mediastinal B‑cell lymphoma who did not receive RT, a localized recurrence should be irradiated.

## Perspective

RT remains an important instrument in the orchestra of treating DLBCL. With the help of diagnostic tools and predictive markers, a further individualized treatment is awaited, enabling a risk-adapted strategy. The development of new targeted therapies challenges the position of RT but also offers the possibility of new synergistic combinations, the analysis of which will be a major task for the radiation oncology community in the years to come.
